# L1 increases adhesion-mediated proliferation and chemoresistance of retinoblastoma

**DOI:** 10.18632/oncotarget.14487

**Published:** 2017-01-04

**Authors:** Dong Hyun Jo, Kyungmin Lee, Jin Hyoung Kim, Hyoung Oh Jun, Younghoon Kim, Young-Lai Cho, Young Suk Yu, Jeong-Ki Min, Jeong Hun Kim

**Affiliations:** ^1^ Fight against Angiogenesis-Related Blindness (FARB) Laboratory, Clinical Research Institute, Seoul National University Hospital, Seoul, Republic of Korea; ^2^ Department of Biomedical Sciences, Seoul National University College of Medicine, Seoul, Republic of Korea; ^3^ Tumor Microenvironment Research Center, Global Core Research Center, Seoul National University, Seoul, Republic of Korea; ^4^ Biotherapeutics Translational Research Center, Korea Research Institute of Bioscience and Biotechnology, Daejeon, Republic of Korea; ^5^ Department of Biomolecular Science, University of Science & Technology, Daejeon, Republic of Korea; ^6^ Department of Pathology, College of Medicine, Seoul National University, Seoul, Republic of Korea; ^7^ Department of Chemistry, Dongguk University, Seoul, Republic of Korea; ^8^ Department of Ophthalmology, Seoul National University College of Medicine, Seoul, Republic of Korea

**Keywords:** retinoblastoma, L1, cell adhesion molecules, adhesion-mediated proliferation, chemoresistance

## Abstract

Retinoblastoma is the most common intraocular cancer in children, affecting 1/20,000 live births. Currently, children with retinoblastoma were treated with chemotherapy using drugs such as carboplatin, vincristine, and etoposide. Unfortunately, if conventional treatment fails, the affected eyes should be removed to prevent extension into adjacent tissues and metastasis. This study is to investigate the roles of L1 in adhesion-mediated proliferation and chemoresistance of retinoblastoma. L1 was differentially expressed in 30 retinoblastoma tissues and 2 retinoblastoma cell lines. Furthermore, the proportions of L1-positive cells in retinoblastoma tumors were negatively linked with the number of Flexner-Wintersteiner rosettes, a characteristic of differentiated retinoblastoma tumors, in each tumor sample. Following *in vitro* experiments using L1-deleted and -overexpressing cells showed that L1 increased adhesion-mediated proliferation of retinoblastoma cells via regulation of cell cycle-associated proteins with modulation of Akt, extracellular signal-regulated kinase, and p38 pathways. In addition, L1 increased resistance against carboplatin, vincristine, and esoposide through up-regulation of apoptosis- and multidrug resistance-related genes. *In vivo* tumor formation and chemoresistance were also positively linked with the levels of L1 in an orthotopic transplantation model in mice. In this manner, L1 increases adhesion-mediated proliferation and chemoresistance of retinoblastoma. Targeted therapy to L1 might be effective in the treatment of retinoblastoma tumors, especially which rapidly proliferate and demonstrate resistance to conventional chemotherapeutic drugs.

## INTRODUCTION

The mainstay treatment options against retinoblastoma, the most common intraocular malignant tumor in children, are chemotherapy and focal therapies [1, 2]. Currently, drug regimens include a combination of carboplatin, vincristine, and etoposide for intravenous administration and melphalan or topotecan for intraarterial administration [3]. If these treatment options fail to manage intraocular tumors, the affected eyes are enucleated to minimize the possibility of extraocular extension and metastasis to other vital organs, including the brain.

Retinoblastoma tumors exhibit varying degrees of differentiation with heterogenous distribution of rosettes, including Homer Wright and Flexner-Wintersteiner rosettes [4, 5]. Retinoblastoma cells which constitute Flexner-Wintersteiner rosettes retain features of primordial photoreceptor cells [4–7]. However, as in other cancers, with the progression of retinoblastoma, retinoblastoma cells lose their ability to differentiate [8, 9] and there was a significant inverse relationship between age at enucleation and the degree of tumor differentiation, demonstrated by the presence of rosettes [6].

L1, a transmembrane glycoprotein, was primarily identified as an adhesion molecule which plays a role in the migration of neuronal cells [10]. Recent studies have demonstrated that L1 is also expressed in several cancers including glioblastoma, neuroblastoma, and pancreatic neuroendocrine tumor [11–15]. In particular, L1 is often linked with poorly differentiated tumors and regarded as poor prognostic factors [13, 16–18]. With homophilic or heterophilic interaction, L1 triggers various signaling pathways [19], resulting in proliferation, migration, invasion, metastasis, and chemoresistance of cancer cells [15, 20–24].

In this study, the roles of L1, which was differentially expressed in human retinoblastoma cells and tumors, were investigated in adhesion-mediated proliferation and chemoresistance of retinoblastoma, using L1-depleted or L1-overexpressing retinoblastoma cell lines and an established orthotopic transplantation model in mice. L1 depletion downregulated the activation of down-stream signaling molecules in phosphoinositide 3-kinase (PI3K)/Akt and mitogen-activated protein kinase (MAPK) pathways, resulting in decreased growth and increased susceptibility to chemotherapeutic drugs. These phenomena might be related with the differential expression of proteins and genes associated with cell cycle, apoptosis, and multi-drug resistance (MDR) by L1. L1 overexpression induced the opposite biological activities in retinoblastoma cells. From these results, we suggest that L1 could be a valuable therapeutic target for retinoblastoma, especially which demonstrates rapid proliferation and resistance to conventional drugs.

## RESULTS

### L1 is differentially expressed in retinoblastoma tissues and cells

**Figure 1:**
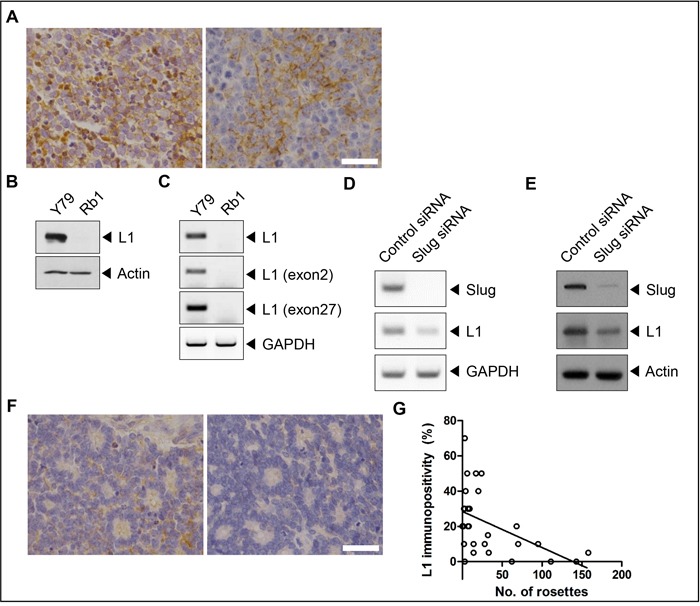
L1 is differentially expressed in retinoblastoma **A**. L1-positive retinoblastoma cells in areas with compactly packed tumor cells in retinoblastoma tissues. **B** and **C**. The expression of L1 in Y79 and SNUOT-Rb1 cells by (B) Western blot analysis and (C) RT-PCR. **D** and **E**. The expression of L1 in control and Slug-depleted Y79 cells by (D) Western blot analysis and (E) RT-PCR. **F**. No to weak immunopositivity of L1 in areas with multiple Flexner-Wintersteiner rosettes in retinoblastoma tissues. **G**. The correlation between the number of Flexner-Wintersteiner rosettes and the proportion of L1-positive cells in each tumor sample. Rb1, SNUOT-Rb1 cells. Scale bar, 25 μm.

As summarized in Supplementary Table 1, 30 retinoblastoma tumors included in this study were representative of advanced retinoblastoma in Group D or E according to International Classification of Retinoblastoma [25] and Group 5A or 5B according to Reese-Ellsworth classification [26]. Immunohistochemical analyses showed that 26 samples (87%) were L1-positive (Figure 1A and Table 1). The mean proportions of L1-positive cells in positive samples were 25% (Table 1). L1 was also found to be differently expressed in two different retinoblastoma cell lines, Y79 and SNUOT-Rb1 cells (Figure 1B).

**Table 1:** L1 expression patterns of primary retinoblastoma tumors (N=30)

L1 positivity	
Positive samples (n, %)	26, 87
Positive cells within each positive sample (%, range)	25, 5-70

L1 is expressed as a neuronal or non-neuronal splicing isoform that demonstrates specific tissue distribution [27–29]. The neuronal isoform of L1 contains exons 2 and 27, whereas the non-neuronal isoform, which is known to be expressed in cancers [30], lacks residues encoded by exons 2 and 27 due to alternative splicing [31–33]. Reverse transcriptase (RT)-polymerase chain reaction (PCR) analyses using specific primers to the exon 2 or 27 showed that L1 which was expressed in Y79 cells was the neuronal isoform containing exons 2 and 27 (Figure 1C). The depletion of Slug, the transcription factor of L1 [34, 35] and increased in retinoblastoma protein-depleted cells [36], decreased the expression of L1 in Y79 cells (Figure 1D and 1E).

To figure out the relation between L1 expression and tumor characteristics, correlation analyses were performed between the proportion of L1-positive cells and the number of Flexner-Wintersteiner rosettes (Figure 1F), a morphological marker of differentiated retinoblastoma [5, 6], in each tissue sample. Interestingly, there was a definite negative correlation between these 2 features (Figure 1G; Pearson's coefficient = -0.493, *P*-value = 0.006).

### L1 increases adhesion-mediated proliferation of retinoblastoma

**Figure 2:**
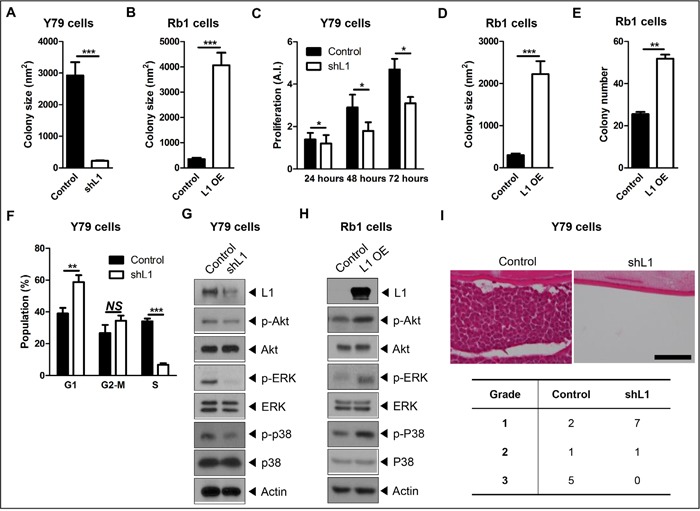
L1 increases adhesion-mediated proliferation of retinoblastoma **A** and **B**. Quantitative analyses of colony formation of control and L1-depleted Y79 cells (A) or control and L1-overexpressing SNUOT-Rb1 cells (B) at 2 days after thawing. **C**. The degree of proliferation of control and L1-depleted Y79 cells at indicated time points. **D** and **E**. Quantitative analyses of colony formation in soft agar in the size (D) and number (E) of colonies. **F**. The distribution of cell cycle of control and L1-depleted Y79 cells analyzed by flow cytometry. **G** and **H**. The expression of L1, phospho-Akt (p-Akt), Akt, phospho-ERK (p-ERK), ERK, phospho-p38 (p-p38), and p38 in control and L1-depleted Y79 cells (G) or control and L1-overexpressing SNUOT-Rb1 cells (H) on Western blot analyses. **I**. Representative photographs of *in vivo* tumor formation at 4 weeks and the proportion of tumors with grade 1, 2, and 3 after intravitreal injection of control or L1-depleted Y79 cells. Scale bar, 100 μm. Control, Y79 or SNUOT-Rb1 cells; shL1, Y79 cells transfected with L1-specific shRNA; L1 OE, SNUOT-Rb1 cells transfected with a lentiviral vector containing full length L1. Bars, SEM. *, *P* < 0.05; **, *P* < 0.01; ***, *P* < 0.001; *NS*, *P* > 0.05 (A-E, Mann-Whitney U-test; F, Unpaired T-test).

To investigate the role of L1 in retinoblastoma, stable retinoblastoma cell lines were established with depletion or overexpression of L1 by lentivirus-mediated transduction of an L1-specific short hairpin RNA (shRNA) or an expression vector for full-length human L1, respectively. As shown in Figure 2A and Supplementary Figure 1A, L1-depleted Y79 cells failed to form cohesive clusters compared to naïve Y79 cells which are characterized by cluster formation in proliferation. In contrast, L1-overexpressing SNUOT-Rb1 cells showed higher cohesiveness compared to naïve SNUOT-Rb1 cells (Figure 2B and Supplementary Figure 1B). Next, to determine the effects of L1 on the proliferation of retinoblastoma cells, the proliferation assays were performed by direct cell counting. L1 depletion significantly decreased the proliferation of retinoblastoma cells in varying serum concentrations (Supplementary Figure 1C) and time points (Figure 2C). In contrast, L1 overexpression increased anchorage-independent growth, as determined by the number and size of colonies in a soft agar assay (Figure 2D, 2E, and Supplementary Figure 1D).

To confirm the effects of L1 on the proliferation of retinoblastoma cells, Y79 cells were synchronized and the cell cycle distribution was examined by flow cytometry analyses. As shown in Figure 2F, L1 depletion induced the accumulation of cells in the G1 phase and subsequent reduction in the entry into the S phase. In accordance with these results, the expression of cyclin D1, E, and A was markedly reduced and that of cell cycle inhibitor p21 and p27 was increased in L1-depleted Y79 cells (Supplementary Figure 1E).

Recent studies have demonstrated that L1 activates intracellular signaling pathways, including PI3K and MAPK pathways, to induce proliferation, invasion, and metastasis of tumor cells [19–22, 37, 38]. In retinoblastoma cells, L1 downregulation resulted in decreased phosphorylation of Akt, extracellular signal-related kinase (ERK), and p38 (Figure 2G), while L1 overexpression induced the increase in activation of these pathways (Figure 2H).

Then, the effects of L1 depletion on *in vivo* tumor formation were investigated with a well-established orthotopic transplantation model in mice. Naïve and L1-depleted Y79 cells (5 × 10^4^ cells) were injected into the vitreous cavity of Balb/c nude mice and the degrees of tumor formation were evaluated according to the visual grading system at 4 weeks after the injection (Supplementary Figure 2). Naïve Y79 cells effectively formed tumors in the vitreous cavity (Figure 2I). In contrast, L1-depleted Y79 cells failed to form mass-like tumors in the vitreous cavity (Figure 2I). In addition, there was a significant difference in the degree of tumor formation, evaluated by the visual grading system, between 2 groups (Figure 2I; Fisher's exact test, *P*-value = 0.013).

### L1 increases chemoresistance of retinoblastoma cells

**Figure 3:**
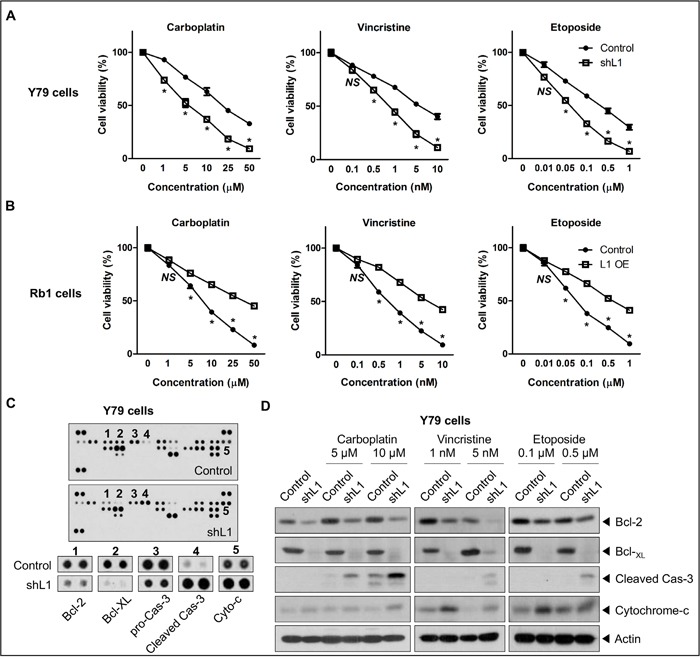
L1 increases chemoresistance of retinoblastoma **A** and **B**. The cell viability of control and L1-depleted Y79 cells (A) or control and L1-overexpressing SNUOT-Rb1 cells (B) upon the treatment with carboplatin, vincristine, and etoposide. **C**. The expression of 35 proteins associated with apoptosis in control and L1-depleted Y79 cells on antibody array. Significantly up-regulated or down-regulated proteins were shown below. **D**. The expression of Bcl-2, Bcl-xL, cleaved caspase-3, cytochrome-c in control and L1-depleted Y79 cells upon the treatment with carboplatin, vincristine, and etoposide on Western blot analyses. Control, Y79 cells or SNUOT-Rb1 cells; shL1, Y79 cells transfected with L1-specific shRNA; L1 OE, SNUOT-Rb1 cells transfected with a lentiviral vector containing full length L1. Cas-3, caspase-3. Bars, SEM. *, *P* < 0.05; NS, *P* > 0.05 (Unpaired T-test).

L1 is known to be linked with protection from apoptosis as well as chemoresistance in various cancer cells [15, 23, 24, 39]. In this context, the relation was investigated between the L1 levels and chemoresistance to carboplatin, vincristine, and etoposide. L1 depletion in Y79 cells significantly decreased cell viability upon the treatment with carboplatin, vincristine, and etoposide (Figure 3A), while L1 overexpression in SNUOT-Rb1 cells increased resistance to these drugs (Figure 3B).

A variety of mechanisms involved in the resistance of cancer cells to chemotherapy include proteins related with apoptosis and MDR [40–42]. To screen which proteins were related with L1-mediated chemoresistance in retinoblastoma, the relative levels of 35 apoptosis-related proteins were analyzed using a protein array in control and L1-depleted Y79 cells. As shown in Figure 3C, pro-apoptotic proteins, cleaved caspase-3 and cytochrome c, were markedly increased, whereas anti-apoptotic proteins, Bcl-2, Bcl-xL, and pro-caspase-3, were reduced in L1-depleted Y79 cells. In accordance with these data, L1 depletion downregulated the expression of Bcl-2 and Bcl-xL and upregulated the expression of cleaved caspase-3 and cytochrome c upon the treatment of carboplatin, vincristine, or etoposide (Figure 3D).

**Figure 4:**
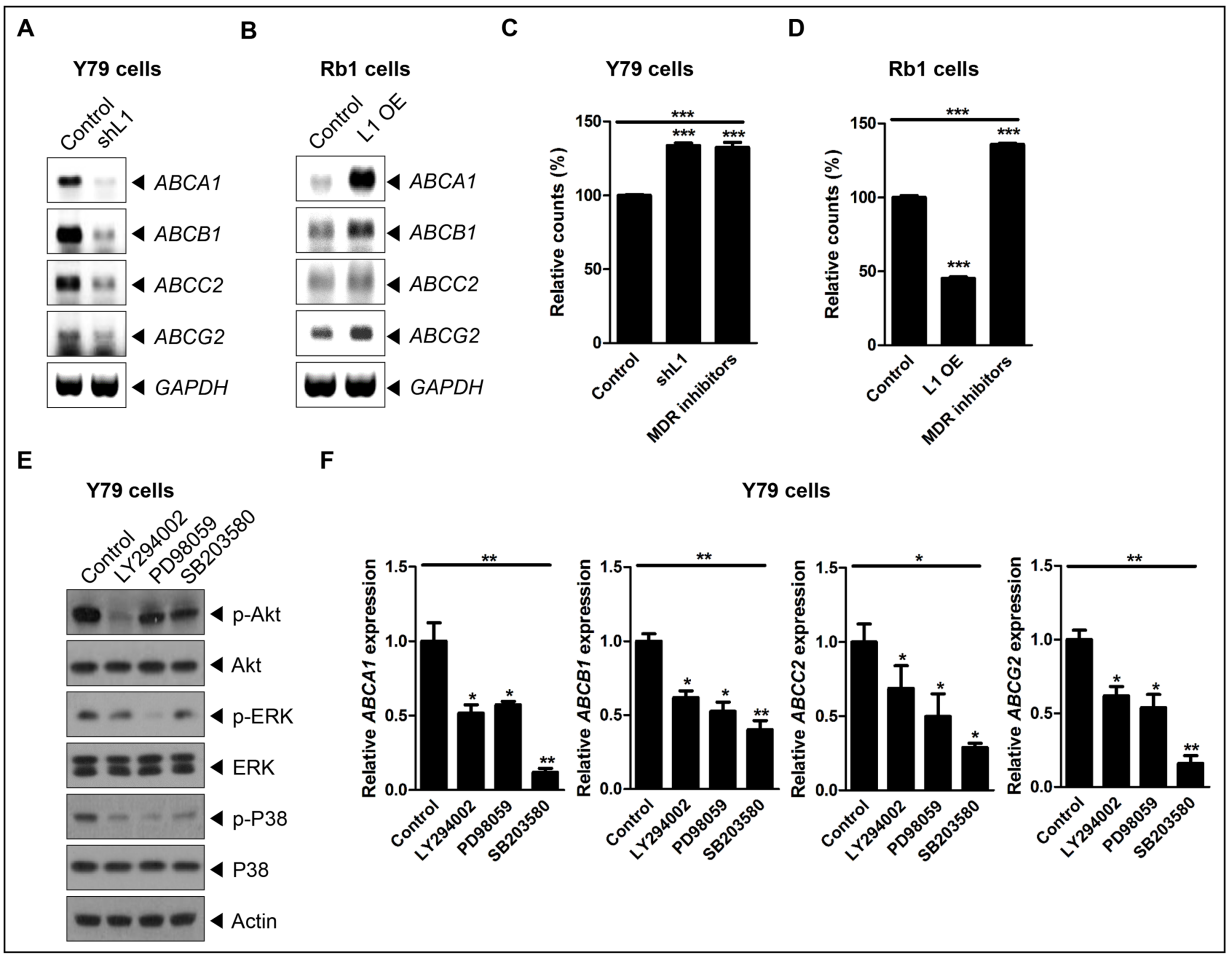
L1 is linked with MDR-related gene expression in retinoblastoma **A** and **B**. The relative expression of *ABCA1*, *ABCB1*, *ABCC2*, and *ABCG2* in control and L1-depleted Y79 cells (A) or control and L1-overexpressing SNUOT-Rb1 cells on RT-PCR. **C** and **D**. The relative fluorescence intensity of control, L1-depleted or -overexpressing cells, and control cells with the treatment of MDR inhibitors on the MDR assay (C, Y79 cells; D, SNUOT-Rb1 cells). **E**. The expression of L1, phospho-Akt (p-Akt), Akt, phospho-ERK (p-ERK), ERK, phospho-p38 (p-p38), and p38 in Y79 cells upon the treatment with LY294002 (an Akt inhibitor), PD98059 (an ERK inhibitor), and SB203580 (a p38 inhibitor) on Western blot analyses. **F**. The relative expression of *ABCA1*, *ABCB1*, *ABCC2*, and *ABCG2* upon the treatment with ERK and p38 pathway inhibitors. Control, Y79 cells or SNUOT-Rb1 cells; shL1, Y79 cells transfected with L1-specific shRNA; L1 OE, SNUOT-Rb1 cells transfected with a lentiviral vector containing full length L1. Bars, SD. *, *P* < 0.05; **, *P* < 0.01; ***, *P* < 0.001 (one-way ANOVA with post-hoc Bonferroni's multiple comparison test).

Next, to investigate whether MDR is involved in L1-mediated chemoresistance of retinoblastoma, the expression of ATP-binding cassette (ABC) transporters were examined in L1-depleted or -overexpressing retinoblastoma cells by RT-PCR and quantitative real-time-PCR (qRT-PCR) analyses. The expression levels of ABC transporters including *ABCA1*, *ABCB1*, *ABCC2*, and *ABCG2* were significantly downregulated in L1-depleted Y79 cells compared to naïve Y79 cells (Figure 4A and Supplementary Figure 3A), whereas those were upregulated in L1-overexpressing SNUOT-Rb1 cells compared to naïve cells (Figure 4B and Supplementary Figure 3B). Consistent with these findings, L1 depletion significantly decreased drug efflux in Y79 cells, comparable to the treatment with MDR inhibitors including verapamil, MK-571, and novobiocin (Figure 4C). In contrast, L1 overexpression upregulated drug efflux in SNUOT-Rb1 cells (Figure 4D).

Several reports have demonstrated that PI3K and MAPK signaling pathways regulate MDR [43–45]. In this context, the expression of ABC transporters was examined with the modulation of Akt, ERK, and p38 pathway in Y79 cells (Figure 4E). As shown in Figure 4E, Akt, ERK, and p38 pathway inhibitors significantly down-regulated the expression of ABC transporters in retinoblastoma cells. Together, these results strongly demonstrated that L1 increased chemoresistance in retinoblastoma.

### L1 increases *in vivo* chemoresistance

**Figure 5:**
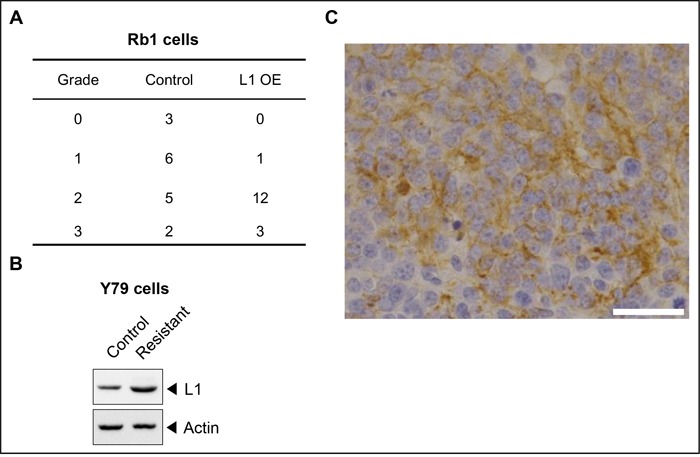
L1 is related with *in vivo* chemoresistance of retinoblastoma and diffusely expressed in chemoresistant human retinoblastoma tumors **A**. The proportion of tumors with grade 0, 1, 2, and 3 on the visual grading system for the orthotopic transplantation model of retinoblastoma in mice. **B**. The proportion of tumors with Flexner-Wintersteiner rosettes in naïve and chemoresistant tumors. **C**. A representative photograph of L1-positive areas in chemoresistant tumors. Control, SNUOT-Rb1 cells; F-W, Flexner-Wintersteiner; L1 OE, SNUOT-Rb1 cells transfected with a lentiviral vector containing full length L1. Scale bar, 25 μm.

Next, to further confirm the effect of L1 on chemoresistance of retinoblastoma *in vivo*, control and L1-overexpressed SNUOT-Rb1 cells (2 × 10^4^ cells) were injected into the vitreous cavity of mice and the mice were treated with carboplatin via intraperitoneal injection 3 times a week from 2 weeks after intravitreal injection of tumor cells, when the tumor formation was evident. Despite vigorous chemotherapy, L1-overexpressing cells formed more mass- (Grade 3) or plaque-like (Grade 2) tumors than control cells (Figure 5A; Fisher's exact test, *P*-value = 0.019). Interestingly, carboplatin-resistant Y79 cells also demonstrated increased L1 expression (Figure 5B). To verify these data using human tumor samples, we performed immunohistochemical analyses in chemoresistant retinoblastoma tumors from 4 patients who underwent multiple cycles of chemotherapy (Supplementary Table 2). As demonstrated in Figure 5C, chemoresistant tumors demonstrated diffuse cytoplasmic and membranous expression of L1 throughout the tumors.

## DISCUSSION

Retinoblastoma is the most common intraocular malignant tumor in children, affecting 1 in 20,000 live births [1, 2, 46, 47]. In the progression of retinoblastoma, retinoblastoma tumors lose their characteristics of differentiated retinal cells [8, 9]. Retinoblastoma tumors from patients who underwent enucleation at older age demonstrate fewer Flexner-Wintersteiner rosettes which represent differentiated retinoblastoma tumors [6]. Electron microscopy studies demonstrate that retinoblastoma cells in Flexner-Wintersteiner rosettes possess cilia, which are thought to be the precursor of photoreceptor outer segments [4, 7]. Although the cell of origin of retinoblastoma is yet to be established [48–50], these features indicate that retinoblastoma cells retain the characteristics of differentiated retinal constituent cells.

In this study, L1 was differentially expressed in retinoblastoma cells and tissues. In 26 (87%) out of 30 human retinoblastoma tumor samples, L1-positive cells were observed with the mean proportions of 25%. Similarly to the differential expression in retinoblastoma tissues, Y79 cells showed higher expression of L1, whereas SNUOT-Rb1 cells lacked expression of L1. Our results also revealed that L1 expressed in Y79 cells was the neuronal isoform containing the neuron-specific exon 2 (YEGHH) and 27 (RSLE) motifs, not the non-neuronal isoform, which is so-called tumor-associated variant [30], lacking both exons (Figure 1D). The L1 variant lacking YEGHH, located at the N-terminus, exhibits significantly reduced hemophilic and heterophilic binding and neurite outgrowth activity [51]. On the other hand, RSLE in the cytoplasmic domain induces the internalization of L1 via clathrin-coated pits [52].

To figure out the meaning of differential expression of L1 in the context of differentiation in retinoblastoma, correlation analyses were performed on the relationship between the proportion of L1-positive cells and the number of Flexner-Wintersteiner rosettes. Interestingly, there was a definite inverse relationship between these 2 features, indicating that L1 might be related with loss of differentiation in retinoblastoma. This finding was in line with previous reports on the expression of L1 in poorly differentiated cancers in other organs [13, 17]. Furthermore, in 4 human retinoblastoma tumor samples from patients who underwent enucleation despite multiple cycles of intravenous chemotherapy, diffuse L1 expression was observed, supporting the role of L1 in the chemoresistance of retinoblastoma. In addition, retinoblastoma tumors, both naïve and chemoresistant tumors, demonstrated mixed cytosolic and membranous expression of L1. These mixed patterns implied that L1 activated intracellular signaling by internalization in retinoblastoma [22], not just remained as an adhesion molecule.

From the functional analyses of L1 in retinoblastoma, L1 depletion induced deactivation of Akt, ERK, and p38 in Y79 cells, whereas L1 overexpression increased phosphorylation of them in SNUOT-Rb1 cells. Both PI3K/Akt and MAPK pathways are downstream signaling cascades of L1 activation in various cancers [20–22, 37, 38]. Furthermore, modulation of these pathways by L1 were linked with differential expression of cell cycle-, apoptosis-, and MDR-related molecules, affecting proliferation and chemoresistance of retinoblastoma.

In conclusion, L1, differentially expressed in retinoblastoma cells and tumors, increases proliferation and chemoresistance of retinoblastoma through the modulation of intracellular signaling cascades, PI3K/Akt and MAPK pathways. Our results support that targeting L1 might provide a new therapeutic strategy that is effective in the treatment of retinoblastoma tumors which differentially express L1 and are resistant to conventional drugs.

## MATERIALS AND METHODS

### Patients

The use of retinoblastoma tissues from 30 patients with retinoblastoma was approved by Institutional Review Board of Seoul National University Hospital (IRB No. 1604-070-754). The demographic and clinical characteristics of 30 patients were provided in Supplementary Table 1. All study protocols conformed to the tenets of the Declaration of Helsinki and the Health Insurance Portability and Accountability Act.

### Animals

6-week-old male Balb/c nude mice were purchased from RaonBio and maintained under a 12-hour dark-light cycle. All animal experiments were performed following the Association for Research in Vision and Ophthalmology statement for the use of animals in ophthalmic and vision research and approved by Institutional Animal Care and Use Committees of both Seoul National University and Korea Research Institute of Bioscience and Biotechnology.

### Cells

Y79 cells (ATCC) and SNUOT-Rb1 cells [53] were maintained in RPMI-1640 (Thermo) with 10% fetal bovine serum (FBS) at 37°C in the humidified atmosphere of 95% air and 5% CO_2_.

### Chemicals

Carboplatin (cat. no. C2538, Sigma), vincristine (cat. no. V8879, Sigma), and etoposide (cat. no. E1383, Sigma) were diluted to indicated concentrations with distilled water (carboplatin), methanol (vincristine), or DMSO (etoposide) for further experiments.

### Immunohistochemistry

From paraffin blocks of enucleated eyes from 30 patients, sagittal sections were prepared at 4 μm thickness. The sections were incubated at 60°C for 2 hours and then deparaffinized and hydrated by sequential immersion in Xylene Substitute (Thermo) and graded ethyl alcohol solutions. Antigen retrieval was performed by the treatment with 0.1M sodium citrate (pH 6.8, Sigma) at 120°C for 10 minutes. The sections were permeabilized with 0.2% Triton X-100 at room temperature for 10 minutes. Then, to minimize nonspecific binding, the sections were treated with 1X Universal Blocking Reagent (Biogenex) for 10 minutes. After incubation with the primary antibody against L1 (1:5000; cat. no. ab24345, abcam) overnight, the sections were treated with REAL™ Detection Systems (Dako) and DAB Kit (Life Technologies) according to the manufacturer's instructions. Then, the sections were mounted with Permount solution (Thermo) and observed under the light microscope (Nikon). The percentage of L1-positive cells in tumor sections were evaluated by an experienced pathologist (Y.H. Kim) and confirmed by another independent observer (D.H. Jo). In addition, the number of Flexner-Wintersteiner rosettes in tumor samples were estimated from direct counting throughout the whole tumor sections.

### Western blot analyses

Equal amount of extracted proteins from cell lysates were separated using 10% SDS-PAGE. The PVDF membranes (Chemicon) which contained transferred proteins was incubated with primary antibodies against Slug (1:1000, cat. no. 9585, Cell Signaling), L1 (1:1,000; abcam), p-Akt (1:1,000; cat. no. 4060, Cell Signaling), Akt (1:1,000; cat. no. 4691, Cell Signaling), p-ERK (1:1,000; cat. no. 9106, Cell Signaling), ERK (1:1,000; cat. no. 9102, Cell Signaling), Cyclin-B1 (1:1,000; cat. no. sc-594, Santa Cruz), Cyclin-A (1:1,000; cat. no. sc-596, Santa Cruz), Cyclin-D1 (1:1,000; cat. no. 2978, Cell Signaling), Cyclin-E (1:1,000; cat. no. sc-247, Santa Cruz), p21 (1:1,000; cat. no. 2947, Cell Signaling), p27 (1:1,000; cat. no. 3686, Cell Signaling), Bcl-2 (1:1,000; cat. no. 2870, Cell Signaling), Bcl-xL (1:1,000; cat. no. 2764, Cell Signaling), cleaved caspase-3 (1:1,000; cat. no. 9661, Cell Signaling), cytochrome-C (1:1,000; cat. no. sc-13561, Santa Cruz), and β-actin (1:5,000; cat. no. abc-2004, AbClone) at 4°C overnight. Then, the membranes were incubated with species-specific peroxidase-conjugated secondary antibodies (1:10,000; cat. no. 31464 (rabbit) and 31430 (mouse), Thermo) at room temperature for 1 hour. Then, they were treated with Amersham ECL Western Blotting Detection Reagent (cat. no. RPN2106, GE) and exposed to X-ray film. The exposed films were scanned using the scanner.

### RNA isolation

Total RNA was isolated using Trizol reagent (Invitrogen) according to the manufacturer's instructions. RNA quality and quantity were determined by ND-2000 Spectrophotometer (Thermo).

### RT-PCR and qRT-PCR

Total RNA was isolated from retinoblastoma cells using the High Pure RNA Isolation kit (Roche). Target RNA was converted to cDNA by treatment with 200 units of reverse transcriptase and 500 ng of oligo(dT) primer in 50 mM Tris- HCl (pH 8.3), 75 mM KCl, 3 mM MgCl2, 10 mM dithiothreitol, and 1 mM dNTPs at 42°C for 1 hour. The reaction was quenched by heating at 70°C for 15 minutes. One microliter of the cDNA mixture was amplified with 50 mM KCl, 10 mM Tris-HCl (pH 8.3), 1.5 mM MgCl2, 0.2 mM dNTPs, 2.5 units of Taq DNA polymerase, and 0.1 μM of each primer. The primers used were 5’-GAGACCTTCGGCGAGTACAG-3’ (forward) and 5’-CTATTCTAGGGCCACGGCAG-3’ (reverse) for whole *L1*; 5’-ATATGAAGGACACCATGTGA-3’ (forward) and 5’-GCAAAGCAGCGGTAGATGCC-3’ (reverse) for exon 2 of *L1*; 5’-TACAGGTCCCTGGAGAGT-3’ (forward) and 5’-GGCCCCTGAGCTGTCATT-3’ (revserse) for exon 27 of *L1*; 5’-TTCCACGCCCAGCTACCCAA-3’ (forward) and 5’-TGGCATGGGGGTCTGAAAGC-3’ (reverse) for *SLUG*; 5’-ATGAGCCGGTCAATGCCCCT-3’ (forward) and 5’-TAGCAGGCCAGCGCTCACAA-3’ (reverse) for *ABCA1*; 5’-TGGTGGCCAGAAACAACGCA-3’ (forward) and 5’-TCACAATGCAGGTGCGGCCT-3’ (reverse) for *ABCB1*; 5’-TGTCCATCCACTGTTTCAAT-3’ (forward) and 5’-AGTTTGGTGGTAGAGGATCT-3’ (reverse) for *ABCC2*; 5’-TCCGTGGTGTGTCTGGAGGA-3’ (forward) and 5’-TGAGCAGGCCCGTGGAACAT-3’ (reverse) for *ABCG2*; 5’-ACACGTTGGCAGTGGGGACA-3’ (forward) and 5’-TGCCTCCTGCACCACCAACT-3’ (reverse) for *GAPDH*. The PCR conditions were as follows: the first denaturation at 94°C for 5 minutes plus 30 cycles of 30 seconds of denaturation at 94°C, 30 seconds of annealing at 50°C, and 30 seconds of elongation at 72°C and a final extension step at 72°C for 10 minutes. Each experiment was performed in triplicate.

The PCR products were electrophoresed on 0.8% agarose gels containing ethidium bromide (Sigma) in a constant 100 V field in RT-PCR. qRT-PCR analyses were performed in a Rotor-gene 6000 thermocycler (Corbett Research) using 1× SYBR Green mix (Invitrogen). Each assay was performed in triplicate, and the mean value was used to calculate the mRNA expression for the genes of interest and the housekeeping reference gene. The amount of the genes of interest in each sample was normalized to that of the reference control.

### Small interfering RNA (siRNA)

Y79 cells were treated with 1 μM siRNA targeting *SLUG* (cat. no. sc-38393, Santa Cruz) using 1 pulse from Neon® Transfection System (Invitrogen) at 1300 V for 20 ms. At 48 hours after the transfection, the cells were prepared for RT-PCR and Western blot.

### Depletion and overexpression of L1

L1-specific and non-target shRNA encoded in pLKO.1 lentiviral vector (Sigma) and full-length human L1 (neuronal isoform) subcloned into a pLVX-EF1α-IRES-Puro lentiviral vector (Clontech) were utilized for depletion and overexpression of L1, respectively. We utilized the neuronal isoform of L1 as a template because Y79 cells expressed neuronal one. To generate stable transfectants, the lentiviral vector was co-transfected into Lenti-293T cells (Clontech) with virus packing mix (Sigma) using Lipofectamine 2000 (Invitrogen) according to the manufacturer's instructions. The virus was added to Y79 cells or SNUOT-Rb1 cells with 5 μg/mL polybrene (Santa Cruz). After 20 hours, media were removed and replaced with fresh media containing 3 μg/mL of puromycin (Santa Cruz). Puromycin-resistant clones were selected by incubating cells in the puromycin-containing media for 2 weeks. L1 expression was analyzed using RT-PCR and Western blot analyses.

### Proliferation assay

Y79 cells (1 × 10^4^ cells) were seeded in 6-well dishes in the RPMI-1640 media. After 72 hours, viable cells were counted with a hemocytometer in triplicates.

### Flow cytometry

Y79 cells (1 × 10^5^ cells) were seeded in 60-mm dishes in the RPMI-1640 media containing 5% FBS. After 24 hours, the cells were harvested and fixed with cold ethanol for 30 minutes. Then, the cells were dehydrated in phosphate-buffered saline containing 2% FBS and 0.1% Tween 20 at 4°C for 30 minutes, centrifuged to the pellet, and resuspended. Then, the cells were treated with RNase (5 μg/mL) at 37°C for 1 hour and stained with propidium iodide and BrdU. The cells were analyzed using FACSCalibur (BD).

### Colony forming assay

To determine anchorage-independent cell growth, 1 × 10^4^ cells (SNUOT-Rb1) were suspended in 3 mL of media containing 0.3% agar with 10% FBS and applied onto pre-solidified 0.6% agar (3 mL) with no FBS in 60-mm dishes. Each experiment was performed triplicate. After 3 weeks of incubation, colonies on soft agar were observed under a phase-contrast microscope. The colony size and number were determined with the Metamorph 7.1 program (Universal Imaging) using the photographs.

### Orthotopic transplation of retinoblastoma cells

Y79 or SNUOT-Rb1 cells were injected into the vitreous cavity of Balb/c nude mice as previously described. To evaluate the effects of carboplatin treatment, carboplatin (50 mg/Kg) was intraperitoneally injected 3 times per week from 2 weeks after the injection of tumor cells. At 4 weeks after the tumor injection, mice were euthanized with CO_2_ inhalation after deep anesthesia. The eyes were evaluated by visual grading and then enucleated for further hematoxylin and eosin staining.

### Apoptosis array

To detect various apoptosis-related proteins simultaneously, equal amount (300 μg) of extracted proteins from cell lysates were prepared for antibody array using Human Apoptosis Antibody Array Kit (cat. no. ARY009, R&D) according to the manufacturer's instructions.

### MDR assay

The MDR functions were analyzed by EFLUXX-ID^®^ Green Multidrug Resistance Assay Kit (cat. no. ENZ-51029-K100, Enzo) according to the manufacturer's instructions. Briefly, single cell suspensions were harvested and counted. Then, equal numbers of cells (5 × 10^5^) were resuspended in RPMI-1640 media containing 5 % FBS with inhibitors or DMSO as diluent control and incubated at 37°C for 10 minutes. Final concentrations of inhibitors are as follows: Verapamil (MDR1 inhibitor; 50 μM); MK-571 (MRP1 inhibitor; 100 μM); Novobiocin (BCRP inhibitor; 100 μM). Green dye was then added and incubated at 37°C for 30 min. Cells were washed once in ice cold complete indicator-free medium before FACS data acquisition using a FACSCalibur (BD). Propidium iodide was used to exclude dead cells from the analysis. Data were analyzed using FlowJo software.

### Statistics

All statistical analyses were performed with IBM SPSS v22.0 (IBM) and were mentioned in figure legends.

## SUPPLEMENTARY MATERIALS FIGURES AND TABLES


