# Supplementary material for: L1 increases adhesion-mediated proliferation and chemoresistance of retinoblastoma
Source: Oncotarget. 2017 Jan 4;8(9):15441–52. doi: 10.18632/oncotarget.14487 (PMC5362498; doi:10.18632/oncotarget.14487)
Supplement: Supplementary file 1 [file oncotarget-08-15441-s001.pdf]

L1 increases adhesion-mediated proliferation and chemoresistance of retinoblastoma

SUPPLEMENTARY FIGURES AND TABLES

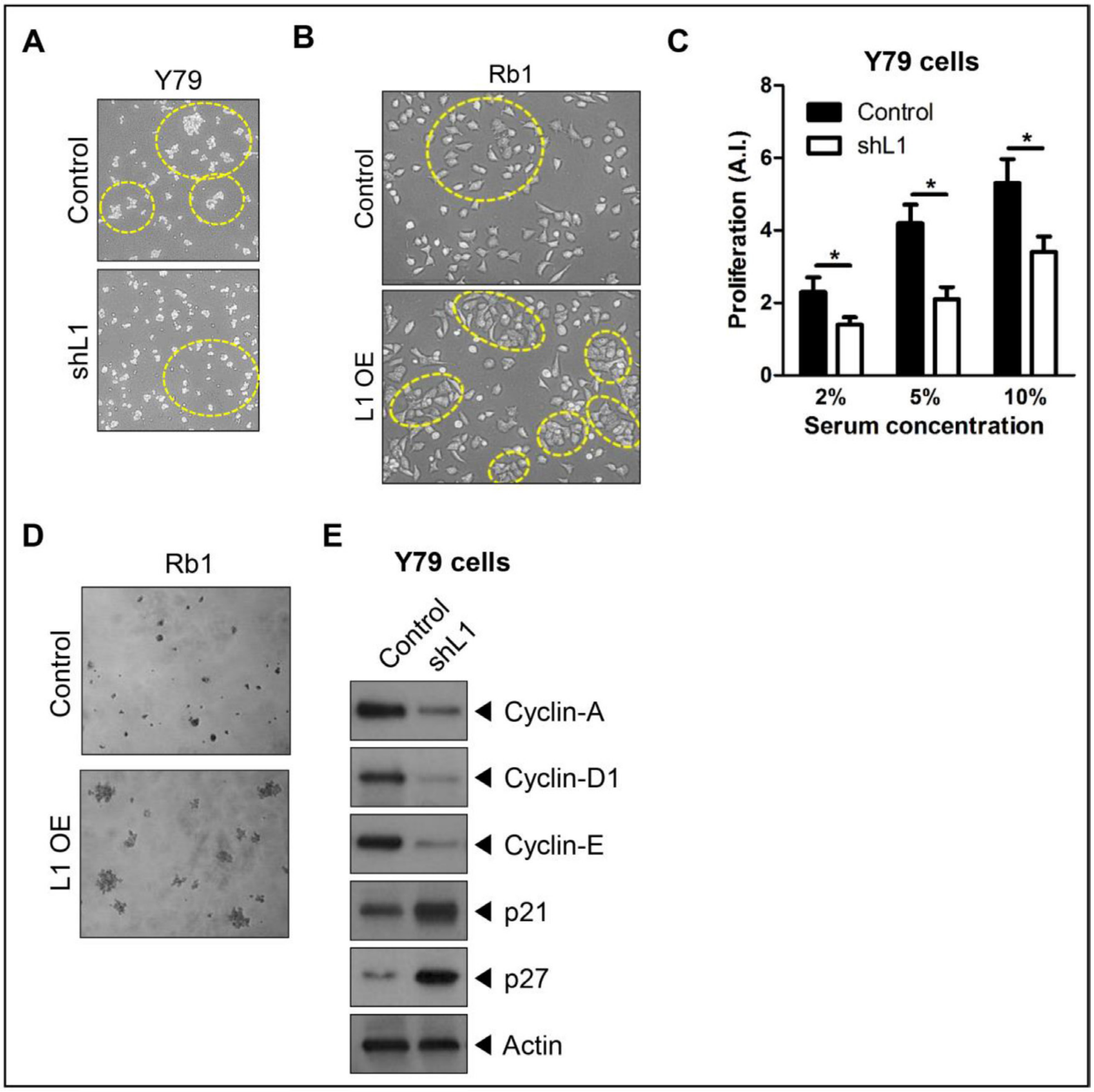

Supplementary Figure 1: L1 increases proliferation of retinoblastoma cells. A and **B.** The morphology of control and L1-depleted Y79 cells (A) or control and L1-overexpressing SNUOT-Rb1 cells (B) at day 2 after thawing using phase contrast microscopy. Dashed yellow circles indicate the clusters of cohesively adherent cells. **C.** The degree of proliferation of control and L1-depleted Y79 cells with different concentrations of serum. **D.** Representative images of anchorage-independent cell growth of control and L1-overexpressing SNUOT-Rb1 cells in soft agar. **E.** The expression of proteins associated with cell cycle in control and L1-depleted Y79 cells on Western blot analyses. Control, Y79 or SNUOT-Rb1 cells; L1 OE, SNUOT-Rb1 cells transfected with a lentiviral vector containing full length L1; shL1, Y79 cells transfected with L1-specific shRNA. Bars, SEM. *, $P < 0.05$ (Mann-Whitney U-test).

Grade	Response to treatment	Specific features	Photo
0	+++	No evidence of tumor	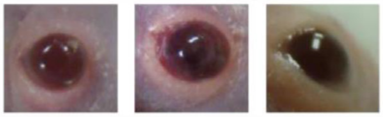
1	++	Vitreous: clear<br>Tumor: streak-like	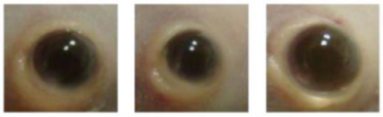
2	+	Vitreous: clear to hazy<br>Tumor: plaque-like	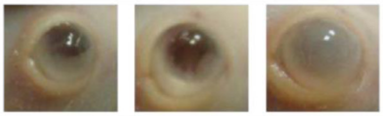
3	+-	Vitreous: hazy<br>Tumor: mass-like	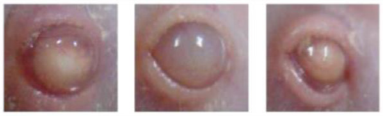
4(4+)	-	Vitreous: uncheckable<br>Tumor: full of vitreous;<br>globe enlargement (4+)	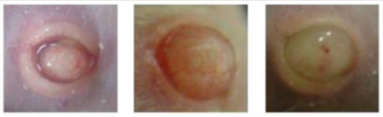

Supplementary Figure 2: A visual grading system for the *in vivo* orthotopic transplantation model of retinoblastoma in mice.

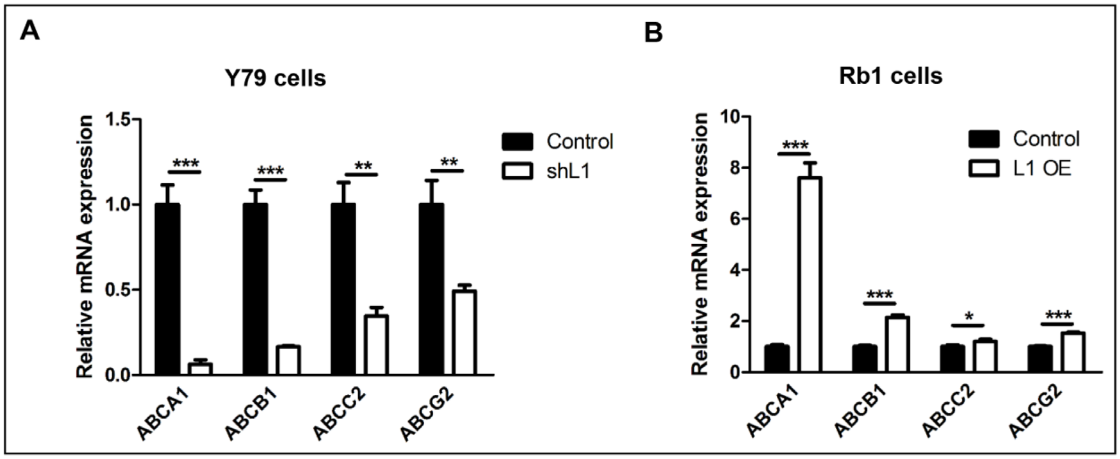

Supplementary Figure 3: L1 increases expression of genes related with MDR. A and **B.** The relative expression of *ABCA1*, *ABCB1*, *ABCC2*, and *ABCG2* in control and L1-depleted Y79 cells (A) or control and L1-overexpressing SNUOT-Rb1 cells (B) on qRT-PCR. Control, Y79 or SNUOT-Rb1 cells; shL1, Y79 cells transfected with L1-specific shRNA; L1 OE, SNUOT-Rb1 cells transfected with a lentiviral vector containing full length L1. Bars, SEM. *, $P < 0.05$; ***, $P < 0.001$; NS, $P > 0.05$ (Unpaired T-test).

Supplementary Table 1: The demographic and clinical characteristics of 30 retinoblastoma patients and tumors in this study

Age at diagnosis (months)	23 ± 17
Female (n, %)	13, 43
Bilateral (n, %)	3, 10
ICR classification	
Group D	11
Group E	19
Reese-Ellsworth classification	
Group 5A	29
Group 5B	1
Tumor size	
Longest diameter (cm)	1.6 ± 0.3
Shortest diameter (cm)	1.0 ± 0.4

Supplementary Table 2: Clinical characteristics of patients with chemoresistant retinoblastoma

Case #	Sex	Chemotherapy regimen
1	F	Cisplatin, etoposide, vincristine #3<br>Ifosfamide, etoposide, vincristine #10<br>Carboplatin, doxorubicin, etoposide #4
2	M	Carboplatin, doxorubicin, etoposide #4<br>Carboplatin, doxorubicin, etoposide, vincristine, cyclophosphamide #10<br>Carboplatin, ifosfamide, etoposide #4<br>Cyclophosphamide, etoposide, topotecan #1
3	F	Carboplatin, doxorubicin, etoposide, vincristine, cyclophosphamide #13
4	M	Carboplatin, doxorubicin, etoposide #2<br>Carboplatin, doxorubicin, etoposide, vincristine, cyclophosphamide #5

#n, number of cycles of indicated chemotherapy regimens.
